# Illuminating neurodegeneration: a future perspective on near-infrared spectroscopy in dementia research

**DOI:** 10.1117/1.NPh.10.2.023514

**Published:** 2023-02-10

**Authors:** Sruthi Srinivasan, Emilia Butters, Liam Collins-Jones, Li Su, John O’Brien, Gemma Bale

**Affiliations:** a University of Cambridge, Department of Engineering, Electrical Engineering, Cambridge, United Kingdom; b University of Cambridge, Department of Psychiatry, Cambridge, United Kingdom; c University College London, Department of Medical Physics, London, United Kingdom; d University of Sheffield, Department of Neuroscience, Sheffield, United Kingdom; e University of Cambridge, Department of Physics, Cambridge, United Kingdom

**Keywords:** near-infrared spectroscopy, dementia, Alzheimer’s disease, functional brain monitoring

## Abstract

**Significance:**

Dementia presents a global healthcare crisis, and neuroimaging is the main method for developing effective diagnoses and treatments. Yet currently, there is a lack of sensitive, portable, and low-cost neuroimaging tools. As dementia is associated with vascular and metabolic dysfunction, near-infrared spectroscopy (NIRS) has the potential to fill this gap.

**Aim:**

This future perspective aims to briefly review the use of NIRS in dementia to date and identify the challenges involved in realizing the full impact of NIRS for dementia research, including device development, study design, and data analysis approaches.

**Approach:**

We briefly appraised the current literature to assess the challenges, giving a critical analysis of the methods used. To assess the sensitivity of different NIRS device configurations to the brain with atrophy (as is common in most forms of dementia), we performed an optical modeling analysis to compare their cortical sensitivity.

**Results:**

The first NIRS dementia study was published in 1996, and the number of studies has increased over time. In general, these studies identified diminished hemodynamic responses in the frontal lobe and altered functional connectivity in dementia. Our analysis showed that traditional (low-density) NIRS arrays are sensitive to the brain with atrophy (although we see a mean decrease of 22% in the relative brain sensitivity with respect to the healthy brain), but there is a significant improvement (a factor of 50 sensitivity increase) with high-density arrays.

**Conclusions:**

NIRS has a bright future in dementia research. Advances in technology – high-density devices and intelligent data analysis—will allow new, naturalistic task designs that may have more clinical relevance and increased reproducibility for longitudinal studies. The portable and low-cost nature of NIRS provides the potential for use in clinical and screening tests.

## 1 Introduction

Currently, 57.4 million individuals live with dementia worldwide with an estimated 152.8 million by 2050,^1^ so there is an urgent need to develop early-stage biomarkers and interventions for dementia. Defined in the International Classification of Diseases (11th Revision) (ICD-11) as an impairment in at least two cognitive domains of sufficient extent to cause impairments in functioning in daily activities, symptoms of dementia include problems with memory, language, executive function, and attention.^2^ The most common form of dementia is Alzheimer’s disease (AD), followed by vascular dementia (VaD), dementia with Lewy bodies (DLB), and fronto-temporal dementia (FTD). Aside from the individual pathophysiology of these disorders, dementia is ultimately associated with large-scale neuronal loss in brain areas that support cognitive function, though earlier cognitive impairment, likely caused by synaptic dysfunction, is thought to occur before such widespread atrophy.^3^ Neither the factors that contribute to this neuronal loss nor the possible targets for intervention are very well established. Early stages in the disease course, such as mild cognitive impairment (MCI) or subjective cognitive decline, may be a critical period for effective intervention in the development of dementia,^4^ in which a conversion rate to dementia of up to 12% a year has been observed.^5^ During this period, pathological changes are proposed to occur in the brain that increase the risk of progression to dementia.^6^ Current methods to detect and monitor dementia, particularly in early prodromal stages such as MCI, are rapidly developing. Recent advances include amyloid and tau brain imaging, the development of cerebrospinal fluid (CSF) biomarkers for tau and amyloid, and most recently blood biomarkers, all of which are available through the National Health Service in the UK. However, many of these biomarkers require validation in early stages, are currently limited to AD, and are not helpful for diagnosing VaD, DLB, or FTD. These biomarkers are also highly invasive, requiring radiation or a lumbar puncture, are expensive, and in the case of CSF and blood measurements, do not directly assess the target organ, i.e., the brain. Given the high misdiagnosis rates observed in dementia, attributed to factors including a lack of resources and attitudes toward diagnosis,^7^ the development of accessible, low-cost, and brain-specific biomarkers is urgently needed.

One promising avenue of research is the relationship between dementia and cerebrovascular and neurometabolic dysfunction as several vascular and metabolic disorders, such as hypertension and diabetes, are associated with later-life cognitive impairment.^8^ Aberrant changes in the vasculature, such as arterial stiffness and increased pulsatility, are thought to lead to neuronal damage in the watershed areas of the main cerebral arteries and are thus strongly associated with cognitive impairment.^9^ Studies using positron emission tomography (PET) have also identified decreased glucose metabolism in several brain regions across dementia subtypes,^10^ in which reduced cerebral metabolism—thought to reflect reduced neuronal activity—is a significant predictor of the conversion from MCI to AD,^11^ although these changes are most likely secondary to neuronal loss and synaptic dysfunction. Additionally, various alterations in functional connectivity (FC) and brain networks have been identified in dementia using functional magnetic resonance imaging (fMRI),^12^ but using these techniques in patient populations can be complicated because they are highly sensitive to movement, are physically restrictive, and have several contraindications. An alternative method to explore vascular dysfunction in dementia is near-infrared spectroscopy (NIRS), a non-invasive neuroimaging technique that measures the hemodynamic response by determining the relative concentration changes of oxygenated (HbO) and deoxygenated hemoglobin (HbR).^13^ NIRS has numerous advantages over traditional neuroimaging methods; it has lower start-up and running costs, is nonionizing, has fewer contraindications, has lower sensitivity to movement, has good temporal resolution, and can be portable, potentially allowing access to people in a wide variety of settings outside the hospital.

**Fig. 1:**
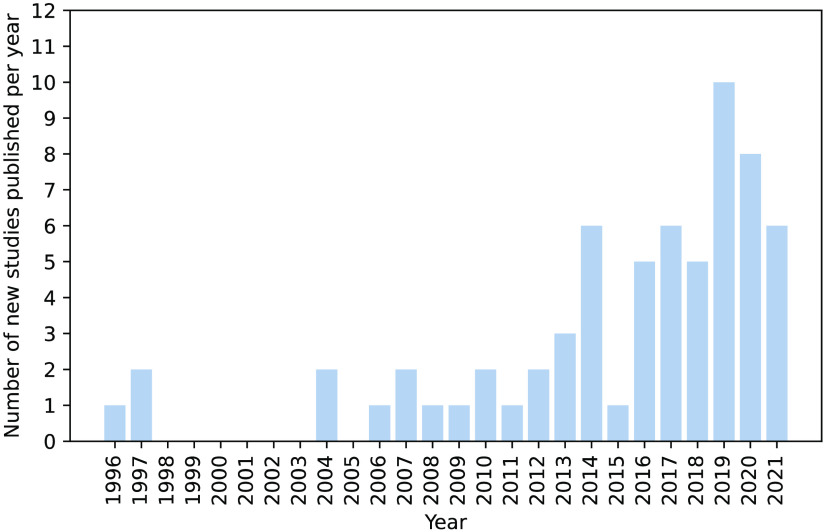
Number of papers published as identified in a search for (“cognitive impairment” OR “cognitive disorder” OR “cognitive decline” OR “vascular dementia” OR “cognitive dysfunction” OR “neurocognitive disorder” OR “Alzheimer*” OR “dement*” OR “AD” OR “memory clinic” OR “FTD” OR “DLB” OR “LBD”) AND (“near-infrared spectroscopy” OR “NIRS” OR “oxyhaemoglobin” OR “tissue oxygenation index”).

The first functional NIRS (fNIRS) studies in humans were published in 1993,^14^ with the first dementia study published in 1996.^15^ Since then, the number of published dementia studies using NIRS has gradually increased (Fig. 1). In general, these studies identified diminished hemodynamic responses localized to frontal regions and widespread disordered FC across a range of cognitive functions in dementia; however, most studies only recorded from the frontal cortex.^16^ There is also evidence for both hypo- and hyper-activation in prodromal disease stages, the latter of which is suggestive of a compensatory response in which alternate brain networks are recruited to counteract neurodegeneration. NIRS has shown promise in being able to detect this compensatory response, and a full systematic review of the results of all studies using NIRS to investigate dementia was previously conducted.^17^ Of these studies, 30 focused on AD, 27 on MCI, 3 on VaD, 1 on FTD, and none on DLB, with 10 studies directly comparing early-stage cognitive decline and later-stage dementia, e.g., Refs. 18 and 19. Most of these studies used dual-wavelength, continuous wave NIRS to measure HbO and HbR changes, with a majority discarding the HbR signal^16^ due to the HbO signal having a higher signal-to-noise ratio and a stronger correlation with the blood-oxygen-level-dependent (BOLD) signal in fMRI. Many studies additionally measured a marker of tissue hemoglobin saturation (or “tissue oxygenation index”) with multidistance NIRS e.g., Refs. 20 and 21. Almost all studies used sparse-channel systems, with only one study^22^ performing diffuse optical tomography (DOT) imaging to produce cortical mappings of task-related activity and none using high-density NIRS systems in conjunction with DOT.

In this paper looking at the future perspectives for the use of NIRS in dementia, we review the challenges involved, from hardware and task design to analysis of data. The full potential of NIRS is not known; for NIRS to become a practical, extensively used tool for dementia research and before we can fully assess it as such, we must address these challenges first and resolve several methodological issues. Here we address these and give suggestions for the progress that needs to be made to maximize the potential of NIRS.

## 2 Improvements in Device Design Increase Cortical Sensitivity and Wearability

The design of the NIRS device itself can dramatically affect the data quality and, therefore, the conclusions drawn from the data. Here, the two major device design aspects, array design and headgear design, are considered, and the implications on the resulting data are discussed.

### 2.1 Array Design

**Fig. 2:**
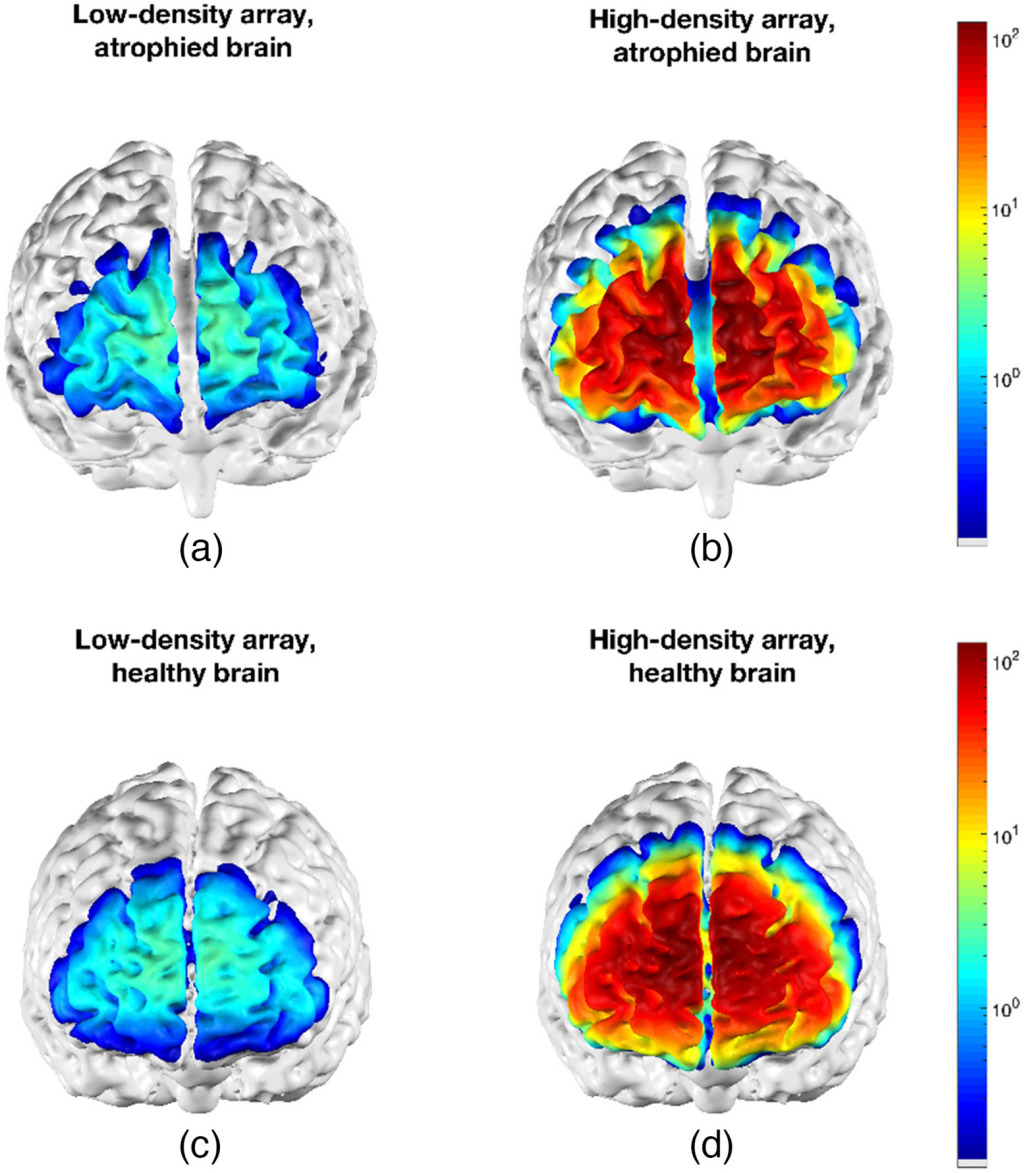
Examples of cortical sensitivity of low-density [(a), (c)] and high-density [(b), (d)] array NIRS on a brain with severe atrophy due to AD [(a), (b)] and a healthy brain [(c), (d)].

Traditional NIRS devices have a sparse design in which a source and detector are placed ∼3  cm apart and the area underneath and between them is monitored. fNIRS assumes that the volume of optical sensitivity includes the brain and therefore that hemoglobin oxygenation changes measured are brain-activity related. For a typical, healthy adult brain, this is likely true. However, in dementia (particularly in AD), the brain can severely atrophy, manifesting as a loss of neurons and neuronal connections (see Fig. 2), meaning that the optical array may no longer be sensitive to absorption changes in the brain.

To ascertain whether optodes placed on the scalp are sensitive to the cortex, we modeled the sensitivity of two different fNIRS arrays using two different head models: one for a healthy adult brain using the Colin27 MRI template^23^ and one for an atrophied brain using MRI data collected from a patient with AD from the Multimodal Imaging in Lewy Body Disorders (MILOS) study (IRAS: 202332). Both MRI datasets were segmented using SPM12^24^ to produce a five-layer head model, which was then converted to a tetrahedral volume mesh using iso2mesh.^25^

To model the state-of-the-art in terms of cortical sampling, we modeled the sensitivity from an array based on the LUMO modular design (Gowerlabs Ltd, London, UK), which we term the *high-density* array. In this high-density array, 12 hexagonal modules (each module containing three sources and four detectors) were positioned on the anterior scalp overlying the frontal cortex/lobe, yielding a total of 36 sources and 48 detectors, with over 400 channels and a source-detector separation in the range 25 to 45 mm. For the *low-density* array, the center positions of each module were taken and assigned as either a source or a detector, forming an array consisting of six sources and six detectors, with the mean nearest neighbor source-detector separation being 30 mm for the dementia head model and 32 mm for the Colin27 head model—a sampling density typical of previous fNIRS research in dementia.

The high- and low-density arrays were registered to each head model. For each array-head model combination (four in total), TOAST++^26^ was used to model near-infrared light propagation from sources to detectors, and the sensitivity to the cortex for each channel was summed to produce a sensitivity distribution.

The results of these simulations are shown in Fig. 2. In both the atrophied and healthy brains, there is a factor of 50 increase in cortical sensitivity using the high-density array compared with the low-density array. There is a similar spatial distribution of cortical sensitivity between the healthy and atrophied brain, particularly in the superior and middle frontal gyri, indicating that high-density arrays are capable of sampling the atrophied brain in this case. (Note that further work needs to be done to assess NIRS brain sensitivity with different types of atrophy in different dementia subtypes and stages and in comparison to age-matched healthy controls.)

However, in the atrophied brain, we see a mean decrease of 22.3% in the relative brain sensitivity of nearest-neighbor channels of the low-density array with respect to the healthy brain. This is comparable to the decrease in sensitivity in the high-density array (reduction in relative brain sensitivity for 25 to 40 mm channels in the high-density array is 20.4%). The decrease in relative brain sensitivity of the low-density array will lead to partial volume effects in which apparent differences in function may be due to changes in anatomy. This is particularly a problem for comparisons between subjects with dementia and healthy controls and for longitudinal studies in which progressive atrophy over time is expected to occur. Crucially, though, the aim of the high-density array is to provide a high level of sensitivity to the cortex, which is demonstrated in the atrophied brain in Fig. 2. This permits an image reconstruction approach to be taken to recover changes in cortical hemoglobin concentration while avoiding the partial volume issues that present substantial limitations for a channel-space analysis of activation in the atrophied brain.

These results highlight the importance of using high-density DOT (HD-DOT). HD-DOT is an imaging technique in which fNIRS data, collected using a high-density array, is combined with a model of light transport—produced using a structural prior of the subject’s head structure—to produce a three-dimensional image localizing hemoglobin concentration changes to the cortex. Overlapping channel measurements (i.e., channels that exhibit sensitivity profiles that partially sample the same volume) in HD-DOT arrays increase the spatial resolution,^27^ improving the precision of functional mapping. A range of source–detector separations allows for depth discrimination, permitting a tomographic approach to study depth-dependent responses, which is important when there is an increased distance of the cortex from the scalp surface due to atrophy. Finally, the inclusion of short separation channels, which predominantly sample nonbrain tissue, enables contamination from scalp hemodynamics to be removed from the cortex-originating functional signal.

Another advantage of DOT is its use of anatomical head structures to model light transport. An anatomically-accurate structural prior increases the accuracy of resulting images,^28^ producing images that are inherently registered to cortical anatomy and enabling improved image interpretation. There is a need for up-to-date, patient-specific data when studying patients whose brains are undergoing atrophy. As can be seen in Fig. 2, there are clear differences in gyrification between the atrophied brain and the brain of a healthy younger adult. Thus, the use of a healthy adult brain for patients with brain atrophy will lead to potential misinterpretations of where activation is localized, so patient-specific structural priors are imperative. Further, given the progressive nature of atrophy in dementia, brain structure will be altered over time, so structural priors derived from a recent MRI scan of a patient are needed. Though MRI scanning is needed to acquire structural data, it will only need to be performed once to allow for multiple HD-DOT scans, such as when performing continuous or longitudinal monitoring over a period (depending on the rate of atrophy).

In the future, it would be ideal to remove the requirement for subject-specific head models. This method is successful in neurodevelopmental NIRS studies of infants in which there are similar challenges in terms of a vulnerable population who are not easy to MRI scan, as well as neuroanatomical challenges that occur between longitudinal measurements. One approach to this would be to produce an atlas of the dementia brain at various stages of progression by averaging structural MRI data taken from many individuals with dementia. Alternatively, another approach is to have a database of head models of subjects with dementia at various stages of progression and with varying head shapes and sizes, and a best matching model can be found for a particular individual based on such characteristics. The challenge moving forward is to systematically determine how to employ a nonsubject-specific head model that minimizes the increase in localization error relative to using subject-specific anatomy.

### 2.2 Headgear Design

Adaptations need to be made to NIRS device designs to improve accessibility for people with dementia. This is a population with reduced mobility, a high probability of having contraindications, and greater frailty. Traditional methods such as computerized tomography (CT) or MRI techniques only provide a snapshot of a patient’s status without accounting for the well-established fluctuations in symptomatology, particularly present in DLB.^29^ As such, devices used to study dementia populations must be able to be worn for continuous monitoring to capture these fluctuations and provide dynamic and richer information of a patient’s vascular state. Devices therefore should also be wearable, robust to movement, portable for use in care homes or at the bedside, comfortable, and easy to use.

### 2.3 Device Conclusion

The results of our optical modeling analysis highlight the importance of using HD-DOT for higher sensitivity, localization of anatomy, and high spatial resolution. Given that optical sensitivity decreases with depth, longitudinal comparisons of brain activity from the same patient with conventional fNIRS may suggest changes in function that are reflective of the patient’s changing anatomy rather than genuine functional changes. Further to this, if high-density or DOT systems are not used, it is imperative that the subject has a recent anatomical (CT or MRI) scan that can confirm that there is no significant atrophy in the region beneath the NIRS optodes. It is also important that a comfortable device is used to increase acceptance in a vulnerable population and improve tolerance for longer, more ecologically valid studies.

## 3 Toward Naturalistic Study Design

Most studies using NIRS in dementia have primarily focused on frontal cortex activation tasks, such as verbal fluency tasks to test word retrieval^30^ and n-back tasks to test working memory function.^31^ In fact, the majority exclusively record from frontal regions, despite AD and DLB predominantly affecting posterior cortices.^32^ Several studies have explored resting state oxygenation via tissue oxygenation index (e.g., Ref. 33), and a handful of studies investigated the influence of symptomatic medication on brain oxygenation (e.g., Ref. 34). Although such studies have shown clear alterations in certain cognitive domains between dementia and healthy aging,^16^ how these alterations relate to clinically relevant outcomes, such as prognosis, treatment response, and potential subgroups, is unclear. A new breed of studies with higher ecological validity are discussed here.

### 3.1 Ecologically Valid Study Designs

As a major advantage of NIRS is its lower sensitivity to movement compared with other neuroimaging methods, dementia patients can be tested during more intensive or naturalistic tasks such as motor tasks; this includes any task that requires speech, as movement of the mouth causes issues for research with both MRI and electroencephalogram (EEG). This is particularly pertinent as many dementia subtypes present with motor deficits, such as DLB, Parkinsonian dementia,^35^ and FTD.^36^ NIRS systems can also be portable and therefore used to perform continuous monitoring in patients’ homes to assess the cognitive fluctuations associated with dementia.^29^ Fiberless NIRS systems offer the ability to study cognitive activity during real-world, naturalistic tasks.^37^ For example, dual-task walking paradigms are often used to investigate the effects of aging on prefrontal activity, e.g., Ref. 38. In the same vein, NIRS systems are highly compatible with virtual reality (VR) systems, enabling the exploration of more naturalistic environments and dynamic conditions.^39^ NIRS studies integrated with VR have been performed to measure prospective memory, which has been shown to be impaired in mild AD patients who were asked to interact with a virtual, immersive town.^40^ Immersive VR settings allow the user to manipulate the virtual environment freely, a feature that can be leveraged to design naturalistic tasks that would provide meaningful insights into memory loss.^41^ These integrated studies offer the potential to perform region-of-interest analyses on a broad range of multimodal data collected during multiple VR task designs, facilitating machine learning (ML) and holistic analysis methods on these augmented datasets.

### 3.2 Functional Connectivity

Although a few studies have explored FC using NIRS (e.g., Refs. 42 and 43), these analyses were done with small channel numbers, low-density systems, or without subject-specific image reconstruction.^44^ Cognitive decline in amnestic MCI (aMCI) and AD is typically addressed using static (spatiotemporally invariant) FC models, in which reduced connectivity is observed in aMCI/mild AD patients.^45^ However, studies utilizing dynamic FC maps have found that the temporal variability of FC is discontinuous in aMCI and AD patients compared with healthy controls.^46^ Subtler alterations in FC may be identified across symptomatology profiles and clinical subgroups, using seed-based approaches and highly detailed topographical maps of brain activation, enabled by HD-DOT.^47^ Additionally, NIRS can easily be compared with other modalities and even used in conjunction with them, such as by combining EEG and NIRS^19^ or PET and NIRS.^48^

### 3.3 Study Design Conclusion

Current fNIRS studies have demonstrated functional dysfunction in dementia, but how this relates to clinically relevant outcomes is yet to be determined. By taking advantage of the wearability of NIRS, improvements in task design with more naturalistic or resting state experiments may allow for the recovery of clinically important biomarkers.

## 4 Need for Standardized, Intelligent, and Automated Data Analysis

One of the biggest challenges in NIRS experiments is the treatment of the data. It is relatively easy to collect data, but to handle it appropriately and draw meaningful conclusions is challenging. We reviewed the current methods used in dementia NIRS studies and look toward a future in which standardized analysis removes subjectivity—potentially with automated, intelligent computing.

### 4.1 Preprocessing

Signal preprocessing is a crucial step in removing noise and extracting useful hemodynamic information from the NIRS data. The vast majority of NIRS studies, including those on dementia, involve similar preprocessing steps. First, raw light intensity signals are converted into changes in HbO and HbR concentrations using the modified Beer–Lambert Law. Physiological sources of noise are commonly removed using a Butterworth bandpass filter with zero-phase filtering to account for phase distortion^49^ and baseline shifts are often eliminated via detrending algorithms (e.g., Ref. 50). Motion correction methodologies vary greatly across NIRS studies in dementia. Most dementia studies exclude trials involving motion artifacts (e.g., Ref. 51), whereas others attempt to perform motion correction using wavelet-based motion artifact removal by decomposing the NIRS signals in the wavelet domain and extracting wavelet detail coefficients (e.g., Refs. 44 and 52).

### 4.2 Traditional Statistical Analysis

Almost all NIRS studies in dementia employ traditional statistical analysis methods. Most commonly, tests of significance using simple statistics, such as t-tests, are used to identify differences in signal metrics across conditions. Activation refers to the increases in relative HbO concentration, and the significance of activation is often determined via per-channel t-tests across patient groups (e.g., Ref. 53). Paired t-tests have been used to compare group mean brain activation levels at different time steps, typically before and after a treatment course or intervention (e.g., Refs. 54 and 55). Analysis of variance (ANOVA) tests, both one-way (e.g., Ref. 56) and two-way (e.g., Refs. 57 and 58), are also often used for group-level comparisons of mean activation or tissue oxygenation index. Correlation analyses between behavioral data, such as the correct answer ratio within tasks, and the degree of brain activation are typically performed using Pearson’s correlation coefficient (e.g., Refs. 59 and 60). When multiple statistical tests are performed, a Bonferroni correction is typically applied to prevent family-wise errors (e.g., Ref. 61).

### 4.3 Machine Learning and Multivariate Analysis

The wide range of neuroimaging modalities used to characterize dementia in the past decades, coupled with nonimaging clinical data from electronic medical records, has led to the generation of large-scale patient datasets.^62^ These large volumes of data can augment traditional methods used to characterize dementia progression through the introduction of ML analysis techniques. ML has a range of applications in dementia research, including predictive modeling of the relationship between input variables and clinical diagnoses, and pattern recognition within the data to study disease progression from MCI to AD.^63^^,^^64^ A large portion of neuroimaging studies use ML to train a model that performs discriminative classification between different patient groups, the most common being classification between AD and healthy controls, but also including classification of MCI from AD and healthy controls, though with generally lower classification accuracies.^65^

Although no dementia research has been performed using HD-DOT and ML, there is a clear opportunity for the high-density recording and localization afforded by HD-DOT to allow for multivariate analysis looking at spatiotemporal dynamics of cortical representations. HD-DOT offers higher spatial resolutions than traditional multichannel NIRS, which lends itself to increased image quality. This offers the possibility of applying convolutional neural networks (CNNs) directly on reconstructed brain images, rather than on statistical representations derived from NIRS channel data (e.g., t-maps).

Both traditional ML analyses and, to a lesser degree, deep learning analyses have been applied to NIRS imaging data to classify between different dementia stages. Finding discriminative NIRS features to classify different stages of AD (mild AD, moderate-severe AD) from healthy controls has been a recent problem, with accuracies around 60% using only NIRS features in multiclass classification, which is lower than with features extracted from other imaging modalities, such as EEG.^19^ In contrast, classification accuracies above 70% have been achieved for binary classification tasks using traditional linear discriminant analysis (LDA) and support vector machine (SVM) classifiers to distinguish between MCI and healthy controls, for example.^66^

CNNs have been trained on spatial and temporal feature maps as input images from both MCI and healthy controls, yielding average classification accuracies of 80% using temporal features and higher classification accuracies using spatial and spatiotemporal features overall.^67^ CNNs have also been trained using both t-maps and correlation maps, achieving classification accuracies of over 90% on binary tasks to classify MCI from healthy controls.^68^ Recently, long short-term memory (LSTM) networks and combined CNN-LSTM models have demonstrated very high accuracies of around 85% on multiclass AD NIRS datasets, compared with a wide variety of traditional ML models, exemplifying the value of applying complex, multilayer models on NIRS data.^67^ The predictive success of the models described above demonstrates the effectiveness of both signal and image biomarkers in the early detection of AD, though further work is required to identify discriminative features to perform diagnosis between less clearly delineated stages of both AD and other forms of dementia.

### 4.4 Data Analysis Conclusion

As is the case for the wider NIRS field, preprocessing (and importantly, reporting of methods) needs to be standardized to ensure consistency across studies and pave the way for multicenter studies with large numbers of patients to produce impactful results. Automation and the use of ML tools, even from the pre-processing stage, may improve the interpretation and understanding of the increasingly complex data captured by NIRS devices.

## 5 Outlook for NIRS in Dementia Research

The existing literature shows that NIRS is a promising tool to study the progression of neurovascular dysfunction in people with dementia. Advances in NIRS device design (particularly wearable HD-DOT) and data analysis have allowed for more complex, ecologically valid experiments, which could improve the clinical relevance of the data. For example, sleep disorders are common in dementia, and wearable NIRS devices allow sleep studies to be carried out in the patient’s home.

Looking further into the future, developments in optical technologies are pushing NIRS beyond hemoglobin oxygenation monitoring. Metabolism is known to decrease as dementia progresses, and it is possible to measure metabolic activity via cytochrome-c-oxidase with broadband NIRS^69^ or via cerebral metabolic rate of oxygen by combining NIRS with diffuse correlation spectroscopy.^70^ It may be possible to measure other dementia-specific parameters, such as amyloid beta deposits in-vivo via NIR fluorescence^71^ or CSF concentration^72^ to observe glymphatic system fluctuations or the level of brain atrophy. The assessment of CSF biomarkers related to neurodegeneration, using broadband NIRS spectral analysis, may also be feasible.^73^ Additionally, optical modeling using time resolved NIRS has demonstrated measurable differences in photon diffusion as a result of CSF thickness variations,^74^ making it a promising method for studying dementia progression. Finally, unique spectral features, identified using broadband NIRS and attributed to the biochemical and structural differences present in those with AD^75^ offer a novel method for investigating the pathophysiology of dementia as a whole. These advances are exciting, and we look forward to the next generation of NIRS studies in dementia.

To conclude, there is a need for suitable neuroimaging tools in dementia research that can capture the dynamic functional, vascular and/or metabolic changes associated with dementia in an unintrusive manner. The recent successes of fNIRS in neurodevelopmental research, in which it is vital to capture subtle longitudinal changes, give promise for the use of NIRS at the other end of the life spectrum - neurodegeneration. We look optimistically to a future in which improvements in the design of NIRS devices and methods have led to fNIRS becoming a well-established and well-regarded tool that dementia researchers and clinicians can use to build an understanding of this complex disease.
